# Clinician views on optimism and empathy in primary care consultations: a qualitative interview study

**DOI:** 10.3399/BJGPO.2021.0221

**Published:** 2022-06-29

**Authors:** Stephanie Hughes, Jane Louise Vennik, Kirsten A Smith, Jennifer Bostock, Jeremy Howick, Christian Mallen, Paul Little, Mohana Ratnapalan, Emily Lyness, Geraldine M Leydon, Hajira Dambha-Miller, Leanne Morrison, Hazel A Everitt, Felicity L Bishop

**Affiliations:** 1 University of Southampton, Primary Care Population Sciences and Medical Education, Southampton, UK; 2 Policy Innovation & Evaluation Research Unit (PIRU): London School of Hygiene & Tropical Medicine, London, UK; 3 University of Oxford, Radcliffe Observatory Quarter, Oxford, UK; 4 Keele University, Primary Care Centre Versus Arthritis, School of Primary, Community and Social Care, Staffordshire, UK; 5 University of Nottingham, Centre for Academic Primary Care, Nottingham, UK; 6 Psychology Department, University of Southampton, Southampton, UK

**Keywords:** Empathy, optimism, Primary Health Care, qualitative research, physician–patient relations, general practitioners

## Abstract

**Background:**

Practitioner expressions of optimism and empathy may improve treatment engagement, adherence, and patient satisfaction but are not delivered consistently amid the challenges of everyday clinical practice.

**Aim:**

To explore primary care practitioner (PCP) views about optimistic and empathic communication in consultations; and to identify behavioural, attitudinal, and/or contextual issues likely to encourage or deter PCPs from practising such communication.

**Design & setting:**

Qualitative interview study with 20 PCPs (GPs, practice nurses, and primary care physiotherapists).

**Method:**

Semi-structured telephone interviews with 20 PCPs. Data were analysed thematically.

**Results:**

A conceptual mismatch between optimism and patient expectations became apparent; when asked how PCPs communicate about the likely effects of a treatment, answers were focussed around managing patient expectations. When prompted, it became clear PCPs were open to communicating optimistically with patients, but emphasised the need for realism. Concerns arose that patients may not be receptive to optimistic messages, especially when holding negative expectations. PCPs felt that expressing empathy is fundamental to all clinical consultations, noting that it can be challenging. Some PCPs worried that increasing expressions of empathy might increase their risk of clinician burnout and felt guilty about (appropriately) communicating empathy while maintaining some emotional distance.

**Conclusion:**

PCPs agreed expressing realistic optimism during consultations could aid communication and would constitute a novel change to practice. PCPs strive for clinical empathy but can struggle to manage emotional self-protection. Specific training to help PCPs express realistic optimism and empathy, and better utilise efficient non-verbal skills could help these issues.

## How this fits in

Clinician optimism and empathy promotes treatment engagement, adherence,and patient satisfaction. Literature suggests there is scope to improve these skills. Through the exploration of clinician views this research identifies important factors for consideration when designing interventions for improving optimism and/or empathy.

## Introduction

Effective practitioner–patient communication within consultations is key to treatment engagement, adherence, and patient satisfaction.^1–3^ Conversely, poor communication can be detrimental to patients in psychological, emotional, social, and economic ways.^4^ For example, consultations in which patient–clinician communication is poor have a 19% higher risk of patient non-adherence to physician recommendations.^2^

Clinicians’ use of positive messages (expressed optimism) and empathy contribute to the effectiveness of communication.^5^ The concepts of clinical empathy and expressed optimism overlap. The most widely used scale for the patient rating of clinician empathy (the CARE measure)^6^ includes the communication of positivity and hope as part of empathy. The effective delivery of positive messages requires a number of components that interlink with empathy, including personalisation, understanding, and trust.^7^ Their intertwined nature makes it helpful to explore these two concepts in tandem.

Clinical optimism, as defined in this study, is the clinician’s expression to a patient of confidence that a suggested plan of action, such as a prescribed medication or a lifestyle change, will likely lead to the desired patient outcomes, such as less pain or better function.^5^ Following clinicians’ expression of optimism, patients may develop positive expectations about the treatment’s effects. In turn this may lead to enhanced effects directly (through placebo-like mechanisms) or indirectly (through changes in patient behaviour).^7^ A positive approach to diagnosis and prognosis can improve patient enablement and reduce symptom burden.^8^ A recent systematic review of randomised controlled trials found that clinician optimism was associated with improved physical symptoms, patient satisfaction, and health-related quality of life.^5^ Much of this evidence derives from pain studies,^9,10^ leaving questions open about the use of optimism in non-pain related consultations.

Many definitions of empathy imply, alongside a sense of understanding, a degree of vicariously experiencing the feelings of another person.^11–13^ Definitions of ‘clinical empathy’ differ slightly, replacing the idea of ‘experiencing’ another’s feelings with the ‘acknowledgement’ of their feelings. The authors interpret clinical empathy as when a clinician *'puts themselves in a patient’s position to acknowledge their feelings, concerns and expectations and behaves in a way to show that they understand'*.^14^ When patients perceive high levels of clinician empathy they experience significantly better long-term outcomes.^3^ This occurs at least in part because empathy helps to motivate and empower patients to self-manage.^15–17^

The evidence suggests that clinical optimism and empathy are important components of the consultation that may enhance patient outcomes, and there may be opportunities to enhance these skills among clinicians.^18–21^ Clinical optimism is a relatively new concept that is not traditionally included in medical school curricula or postgraduate training, and empathy has been subsumed into generic communication and consultation skills training courses for students and practising clinicians. There appears to be little information available about the extent to which a distinction is made between empathy and clinical empathy in current training. New educational resources could help clinicians to acquire, enhance, and embed the skills needed to regularly communicate clinical empathy and optimism in practice; to be engaging, such resources should address PCPs' perspectives. This study, therefore, aimed to explore PCPs' views on conveying optimism and empathy in consultations. The objective was to identify barriers and facilitators to incorporating these elements of communication into consultations. The findings will inform the development of a digital training package for PCPs aiming to improve clinician communication skills in clinical empathy and optimism, in order to improve patient outcomes.

## Method

### Design, setting, and participants

This study used semi-structured telephone interviews.

Ethical approval for the study was granted by a local NHS Research Ethics Committee. The Wessex Clinical Research Network provided the researchers with a list of potential participants, from which the authors purposively sampled male and female individuals with a range of ages and experience levels. Twenty PCPs from 17 GP surgeries in southern England were recruited. Practice list sizes varied from 6000–21 000 (6000–11 000, *n* = 8, 40%; 11 001–16 000, *n* = 5, 25%; 16 001–21 000, *n* = 7, 35%). Table 1 shows detailed characteristics.

**Table 1.** Table of characteristics

Characteristics		*n*	%
Sex			
Male		11	55
Female		9	45
Role			
GP partner^a^		15	75
Salaried GP^b^		1	5
Nurse practitioner^c^		2	10
Primary care physiotherapist^d^		2	10
Ethnicity			
White British		17	85
Other White		1	5
Asian		1	5
Other mixed background		1	5
Age, years			
31–40		7	35
41–50		8	40
51–60		5	25
Experience as a GP/nurse/physiotherapist, years			
≤5		4	20
6–10		1	5
11–15		7	35
16–20		3	15
>20		5	25
Social deprivation scores of practices^33^			
1–2	(High deprivation)↑↓(Low deprivation)	1	5
3–4		3	15
5–6		1	5
7–8		3	15
9–10		12	60

a Principle GP who owns a share of the business and is self-employed.

b GP employed by a GP practice or primary care trust.

c A registered nurse with a range of additional generalist skills providing care for patients with both acute and long-term conditions.

d First contact physiotherapist based in primary care (patients may have self-referred or been directed to the physiotherapist without medical review).

### Data collection and analysis

Semi-structured telephone interviews lasting 26–71 minutes were conducted by three researchers experienced in qualitative interviewing (SH, JV, and KS). Recordings were transcribed verbatim, identifiable participant information was removed, and a unique study ID assigned to each participant to provide anonymity. A topic guide (see Supplementary appendices) was used flexibly to allow for expansion or deeper exploration of relevant topics. Interviewers worked collaboratively to iteratively amend the topic guide where appropriate throughout the process of data collection. For example, initially PCPs were asked early in the interview to describe a recent consultation where communication had gone well, but after a few interviews this question was moved to after questions on empathy and optimism. This change in context resulted in richer data that was more relevant to the research objectives. Drawing on the primary care expertise within the team, the questions designed to tease out thoughts on optimism were framed around patient expectations. This was done because the term ‘patient expectations’ is familiar to PCPs and the authors wanted to generate open, broad discussion and explore whether PCPs mentioned optimism without prompting. Broad questions about patient expectations were followed with specific questions about optimism.

Transcripts were read multiple times to achieve familiarity and were coded in NVivo (version 11). A coding manual was developed after inductively coding five transcripts, was amended iteratively throughout coding the whole dataset, and documented in an audit trail. SH led the analysis with input from the multidisciplinary research team, which incorporates perspectives of health psychology, general practice, sociology, philosophy, human–computer interaction, and patient contributors. Transcripts were analysed using inductive thematic analysis^22^ incorporating techniques from grounded theory^23,24^ (see Supplementary Table 1 in appendices for the analysis process). This study looked for, but did not identify, major differences between GPs, physiotherapists, and nurse practitioners on the qualitative themes.

## Results

The interviews revealed four main areas of interest: an apparent conceptual mismatch between optimism, positive messages, and managing patient expectations; challenges and facilitators around communicating optimism in consultations; PCPs conceptualisation of empathy within a clinical context; and PCP thoughts about demonstrating empathy within clinical consultations.

### A conceptual mismatch: optimism, positive messages, and managing patient expectations

While exploring the use of optimism in the consultation a conceptual mismatch became apparent. The authors wanted to explore how PCPs may encourage optimism in patients, to ultimately improve outcomes. The authors asked the participants how they communicate with patients about the likely effects of a treatment, how they discuss patient expectations, and what they think about the idea of encouraging patients to be optimistic about their treatment outcomes. However, PCPs focused on unrealistic patient expectations (for example when patients expect to be completely cured), and the need to manage these by being realistic and clear. For example, when asked '*how do you communicate with patients about the likely effects of a management strategy, therapy, or treatment?*' one PCP responded:

*'I certainly try never to give absolutes — or to give sort of — undue or unrealistic expectations. So I certainly try to dampen down expectations of cure, for example, in osteoarthritis and empathise about the condition to live with, as opposed to a condition to sort of get rid of, as such.' (P0101, GP partner, 10 years' experience*)

Clinical optimism did not appear to be at the forefront of PCPs minds when describing the way they communicate, however, when prompted they agreed that showing optimism about treatment plans would be a novel and potentially useful approach for encouraging engagement with PCP advice.

### Communicating optimism in consultations: challenges and facilitators

Talking positively and staying upbeat were viewed as effective clinical optimism methods to promote patient optimism:

*'yes, I think being positive and saying, look, you know, we can do something about your pain. I think it’s an important message because they do pick up on what the doctor’s mood is about it and whether they’re dismissive or — some may think it’s worth a try,' (P0107, GP partner, 15 years’ experience*)

By far the biggest barrier to communicating optimism was PCPs’ perceptions of patients’ rigid and unrealistic expectations for an easy fix or cure that requires no work on their part:

*'Some patients have an expectation that things should just be put right and so they find it very difficult if they’ve got — if their expectations aren’t met or that they’ve got to engage with things more before they are going to be met.' (P0115, GP partner, 15 years’ experience*)

A lack of time was described as presenting a challenge in negotiating patient expectations and promoting optimism in the proposed treatment plan within a 10-minute consultation. Other barriers to staying optimistic included a history of failed attempts at pain management, longevity of pain, and patient distress. PCPs expressed concern that being optimistic can sometimes sound patronising and uncaring, and some worried that showing optimism may come across as minimising patient distress. When asked '*are there times you think being optimistic, encouraging optimism, might be inappropriate*?' one PCP replied:

*'Yes. I think you need to — I think definitely if you are optimistic and the patient is clearly in a lot of distress, it may sound patronising and — and uncaring. If they’re — if all they hear is you saying optimistic* [things]*, they may feel that you’re actually not really appreciating the degree and severity of their symptoms,' (P0110, GP partner, 15 years’ experience*)

### Conceptualising (clinical) empathy in clinical consultations

PCPs readily conceptualised empathy as a valuable, vital part of every clinical consultation. While they did not use the term ‘clinical empathy’, PCPs alluded to key features of clinical empathy by talking about how they tried to imagine themselves in their patient’s position and to convey an appreciation of the broader impact of health concerns on the individual person, for example:

*'I try and — and do a lot of acknowledging; so, you know, the sort of — I can imagine that’s really difficult, or — some sort of direct or couple of words, just to say — I’m thinking about, you know, basically I’m saying I’m thinking about — imagining what it’s like to be you or to have that problem or whatever and I can imagine that isn’t very nice,' (P0101, GP partner, 10 years' experience*)

There was some evidence in PCPs’ talk of conceptual overlap with everyday notions of empathy entailing a deeper emotional involvement that is not required for clinical empathy. On the one hand, PCPs expressed concerns that empathy can at times appear fake without this emotional involvement, but on the other hand, they acknowledged that clinicians actually feeling what the patient is feeling can contribute to clinician burnout. In this way, empathy was conceptualised as a limited personal resource at risk of being depleted if not carefully rationed. This tension between wanting to avoid ‘fake’ empathy, while also protecting oneself was managed by distinguishing between empathy and sympathy,^1^ and conceptualising ‘enacted empathy’.^2^ While PCPs may feel they have a ‘limited pool’ of empathy, they discussed having the capacity to display sympathy freely, requiring less clinician emotional investment, thus protecting themselves from burnout. ‘Enacting’ empathy without emotionally investing in the patients’ problems (which is consistent with concepts of clinical empathy) was seen as protective but did not always feel comfortable morally:

*'Well, I think there’s a difference between genuine empathy and feigned empathy. So, you can act, if you're doing consultations you are, for the most part, an actor but you obviously are injecting some of yourself into that as well and that perhaps the more you get to know your patients, the more of yourself you inject into the consultation. I think, in terms of genuine empathy, yes, I think you do have a limited pool of that and I don't think you can use that, but I think that it is worth saying again that sympathy, I try and have sympathy for all my patients and I suppose in a way acting the empathy to all of them and then for certain cases, not — then genuine empathy. That sounds horrible, doesn't it?' (P0120, GP, 3 years’ experience*)

### Demonstrating empathy within consultations: challenges and facilitators of clinical empathy

PCPs were very familiar with techniques for conveying empathy in consultations and could readily describe techniques including building rapport, using open-ended questions, refraining from the use of jargon, making eye contact, adjusting their tone of voice, and mirroring patients’ body language:

*'Communication skills-wise, I’ll be trying to adjust my voice to — and posture — to theirs, mirroring, you tend to be in a similar sort of way of sitting, to them. And — I would be asking at the end — is there anything else I haven’t covered? Checking that they are okay,' (P0113, GP partner, 28 years’ experience*)

These techniques form the foundation of good clinical communication, contributing to the demonstration of clinical empathy. According to PCPs, empathy is enacted by demonstrating an understanding of the broader implications of a patient’s condition on their day-to-day life, for example, how it affects pursuing hobbies, going to work, or interacting with friends and family. Intertwined with this behaviour was showing an interest in the patient’s concerns and motivations:

*'I think empathy would be trying to frame symptoms from their personal perspective … trying to frame it within their psychosocial context for the patient; so impact on their day-to-day living on things such as work, what support network they might have around them, family, friends, you know, things like sports, sleep, mood, how their symptoms might be affecting them on that basis.' (P0110, GP partner, 15 years’ experience*)

The PCP’s perceptions of patient’s characteristics, cognitive, and emotional mindset, and behaviour impacted the ease with which they felt able to express empathy. PCPs described how expressing empathy feels easier and more natural when patients have symptoms that the PCP has experienced, when patients are ‘nice’, and are willing to help themselves:

*'if they seem like they need — that they need and want to be helped ... then that would trigger it* [empathy]*, I suppose,' (P0101, GP partner, 10 years’ experience*)

PCPs described finding it difficult to express empathy to patients who: begin the consultation demonstrating anger or frustration; come in wanting a specific treatment that is not clinically appropriate for them and/or is not accessible through the NHS; have consulted multiple times about the same issue without following the PCP’s advice; or are not seeming to make efforts to improve their own health:

*'I find it hard to empathise with people who won’t help themselves. All doctors must have these patients, but there are some people who you help, those sorts of people who just won’t do anything to — won’t engage with any suggestions and they just keep coming in with the same complaints, having done none of the things you suggested,' (P0114, GP partner, 3 years’ experience*)

Other more practical factors can also stand in the way of empathy, for example, language barriers, cultural differences, or the presence of additional relatives (such as children) at the consultation. PCPs also felt that a lack of time limited their ability to express empathy, as could their own mood and personal situation:

*'I think it’s not necessarily the patient and the problem with the patient, but more — but more how I’m feeling, so that definitely plays a role,' (P0103, GP partner, 13 years’ experience*)

## Discussion

### Summary

Eliciting patient expectations, remaining honest and realistic, and displaying empathy are high on the PCP agenda in consultations, but are not always easy to deliver in practice. The idea of being optimistic in consultations and delivering positive, yet realistic, messages about treatment outcomes, where clinically appropriate, was not mentioned by PCPs unless prompted, suggesting this notion was less familiar.

The concept of empathy was well understood by PCPs, and all agreed it is fundamental in clinical consultations across diverse presentations, even though expressing empathy is not always easy. Fear of burnout, lack of time, the perception that patients ‘do not help themselves’, and other personal factors such as patient/PCP mood or contextual circumstances may hinder expressions of empathy.

The PCPs’ description of a ‘limited pool’ of empathy suggests PCPs understand empathy as a limited personal resource in danger of depletion. To reduce emotional investment and preserve empathy reserves, PCPs describe instead using ‘sympathy’, sometimes with guilt that ‘true’ empathy was not being offered. In fact, what was described falls under researchers’ definitions of clinical empathy, but with some emotional self-protection (see below). The differences between sympathy and empathy have been widely discussed in previous literature; sympathy *‘consists of feelings of sorrow or concern for another’.*^25^ In a healthcare setting, displaying empathy goes further than showing sorrow or concern for a patient and involves demonstrating an understanding of the patient’s perspective, how the illness impacts the patient’s life, and patient–practitioner shared decisionmaking.^3^

### Strengths and limitations

Interviews and analysis were conducted rigorously by a team of experienced qualitative researchers. To minimise idiosyncratic interpretations, the multidisciplinary team were consulted throughout the analytical process to ensure consistency and agreement within data interpretation. In addition, 10% of interview transcripts were double coded and codes were compared for consistency.

While PCPs were purposively sampled and included a good mix of age, experience, and sex, the majority of the participants (*n* = 15/20) were GP partners and White British (*n* = 17/20), and all were recruited from GP surgeries in southern England. A wider range of ethnicities, an expanded geographical location, and more even distribution of PCPs, for example salaried GPs, physiotherapists, and practice nurses may have provided a more diverse range of perspectives. Participation was voluntary; possibly the study appealed to those with a particular interest in communication, thus results may differ with a non-self-selecting sample.

### Comparison with existing literature

The use of positive messages within clinical consultations have been shown to have small, but important benefits to clinical outcomes.^5^ The results of this study indicate that while clinicians are interested in the idea of providing positive messages, work is needed to encourage them to prioritise this approach.

Previous research has explored the tension between the clinician’s desire to remain detached to their patients’ personal emotional situation and the patients’ desire for genuine empathy.^26^ The definition of ‘clinical empathy’ from the Society for General Internal Medicine acknowledges the clinician’s need to remain detached and defines empathy as '*the act of correctly acknowledging the emotional state of another without experiencing that state oneself*'.^27^ The results of this study reflect this and suggest ‘enacting’ empathy without emotionally investing in the patients’ situation is an important and necessary self-protective measure. Clinicians should be encouraged not to feel guilty when expressing empathy and simultaneously employing emotional self-protection.

The concept of enacting empathy in clinical consultations has been explored previously.^28,29^ Hardman *et al* ^29^ identified a pattern in which clinicians ‘*intuitively develop a meta habit of enacting*’ throughout consultations.^29^ Displaying clinical empathy has been described as *‘emotional labor’* requiring *‘deep acting’* and *‘surface acting’*.^28^ Larson *et al* ^28^ distinguish between the two; deep acting is superior to surface acting and involves clinician personal emotional understanding and investment, whereas surface acting can be a substitute when the clinician’s ability to provide genuine emotional understanding is limited.^28^ The findings of this study suggest PCPs conduct ‘deep acting’ when it is necessary and possible, and revert to ‘surface acting’ (referred to by the participants as ‘sympathy’) when emotional resources are limited.

The current findings suggest PCPs view empathy as a limited resource in danger of depletion, and they reserve genuine empathy for those most in need, or most ‘worthy’. These views align with philosophical perspectives arguing that our natural ability to express genuine empathy is easier, and thus depleted, within our immediate social circles, or those who we perceive as similar to ourselves.^30^ Defining empathy as a limited resource used in specific settings means that only a small supply is left for those outside our inner circle.^30,31^ The ethical implications of this uneven investment suggests there is a risk '*empathy pushes partiality into prejudice'* ^32^ and although the authors found no evidence of this, further research may be warranted. Helping PCPs to conceptualise clinical empathy and to distinguish between this and everyday empathy may be useful for addressing the tensions between emotional investment and burnout.

### Implications for research

The findings of this study show that PCPs may be interested in communicating optimism, where appropriate and realistic, within clinical consultations. Further work is needed to examine specific ways in which this could be achieved.

Further research into the notion that individuals have a limited supply of empathy specifically within the context of clinical empathy and clinical consultations may generate ideas to limit empathy bias. The current research revealed barriers to expressing empathy, including a description of the groups of patients where clinicians found it more difficult. Work to develop and test techniques for overcoming these barriers would be a useful next step.

This study provides a detailed insight into PCPs’ views on optimism and empathy and highlights the barriers and opportunities for enhancing communication in everyday clinical practice.

It would be beneficial for future training in optimism and empathy to help PCPs conceptualise optimism and its relevance to clinical practice, help PCPs to distinguish between empathy, sympathy, and clinical empathy, and to acknowledge and address the barriers to communicating empathy and optimism identified in this study.
